# Enhancing Efficiency and Stability of Photovoltaic Cells by Using Perovskite/Zr‐MOF Heterojunction Including Bilayer and Hybrid Structures

**DOI:** 10.1002/advs.201801715

**Published:** 2019-01-01

**Authors:** Chia‐Chen Lee, Chih‐I Chen, Yu‐Te Liao, Kevin C.‐W. Wu, Chu‐Chen Chueh

**Affiliations:** ^1^ Department of Chemical Engineering National Taiwan University Taipei 10617 Taiwan; ^2^ Advanced Research Center for Green Materials Science and Technology National Taiwan University Taipei 10617 Taiwan; ^3^ Center of Atomic Initiative for New Materials (AI‐MAT) National Taiwan University Taipei 10617 Taiwan; ^4^ International Graduate Program of Molecular Science and Technology (NTU‐MST) National Taiwan University Taipei 10617 Taiwan

**Keywords:** heterojunctions, metal–organic frameworks, perovskite, solar cells, stability

## Abstract

In this study, the effectiveness of using a perovskite/Zr‐metal–organic frameworks (MOFs) heterojunction in realizing efficient and stable inverted p–i–n perovskite solar cells (PVSCs) is demonstrated. Two types of Zr‐MOFs, UiO‐66 and MOF‐808, are investigated owing to their respectable moisture and chemical stabilities. The MOFs while serving as an interlayer in conjunction with the perovskite film are shown to possess the advantages of UV‐filtering capability and enhancing perovskite crystallinity. Consequently, the UiO‐66/MOF‐808‐modified PVSCs yield enhanced power conversion efficiencies (PCEs) of 17.01% and 16.55%, outperforming the control device (15.79%). While further utilizing a perovskite/Zr‐MOF hybrid heterojunction to fabricate the devices, the hybrid MOFs are found to possibly distribute over the perovskite grain boundary providing a grain‐locking effect to simultaneously passivate the defects and to reinforce the film's robustness against moisture invasion. As a result, the PCEs of the UiO‐66/MOF‐808‐hybrid PVSCs are further enhanced to 18.01% and 17.81%, respectively. Besides, over 70% of the initial PCE is retained after being stored in air (25 °C and relative humidity of 60 ± 5%) for over 2 weeks, in contrast to the quick degradation observed for the control device. This study demonstrates the promising potential of using perovskite/MOF heterojunctions to fabricate efficient and stable PVSCs.

## 1 Introduction

Organic–inorganic hybrid perovskite has recently attracted significant research attention in the photovoltaic community owing to its facile solution processability and exceptional optoelectronic properties.^1^, ^2^, ^3^, ^4^, ^5^, ^6^ In the past few years, remarkably semiconducting properties of the perovskite materials have been gradually identified, including intense wide‐range light‐harvesting, long carrier diffusion length, and tunable bandgaps, rendering them as an outstanding photovoltaic materials.^7^, ^8^, ^9^, ^10^ With these fundamental understandings, the power conversion efficiency (PCE) of perovskite solar cells has achieved an impressively rapid progress since its first debut in 2009.^11^ At present, the record PCE of perovskite solar cells (PVSCs) has reached 23.2% in this year.^12^, ^13^ This performance rivaling the value of the existing photovoltaic techniques shows great potential for commercialization due to its low‐cost and light‐weight advantages.^14^, ^15^

In order to realize the commercialization, most of the current researches pertaining to PVSCs are mainly focused on improving device's long‐term stability. Many studies have manifested that the moisture in the environment will beget the irreversible degradation of perovskite because H_2_O will form H‐bonding with the constituent ions of perovskite to make the lattice collapsed.^16^, ^17^ On the other hand, intense thermal/photostresses might engender the iodide oxidation to result in the formation of I_2_ and the volatilization of CH_3_NH_2_, causing material degradation.^17^, ^18^ More recent studies have unveiled that such inferior moisture/thermal/photostability is closely related to the defective states of the solution‐processed perovskite film because its polycrystalline nature will unavoidably accompany the formation of imperfect grain boundaries, providing pathways for the external stresses to incur degradation.^19^ To date, significant efforts have been devoted to improve the quality and crystallinity of the perovskite film or alter its surface energy or texture, especially through the composition modulation or using functional additives, to improve its robustness against the stresses.^20^, ^21^, ^22^ Besides reinforcing the intrinsic stability of the perovskite materials, employing proper interlayers is another effective strategy to improve device's long‐term stability as demonstrated in the literatures.^23^, ^24^

Based on similar rationale, we herein propose to modify the properties of the perovskite film using metal–organic framework (MOF)/perovskite heterojunction, given the superior moisture and chemical stabilities of MOF and its scarce investigations until now. As known, MOFs are 3D porous crystalline materials consisting of multipodal organic linkers, like terephthalic acid and trimesic acid, and secondary building units (SBUs) based on high‐valent ions/clusters, like Zr^4+^/Zr_6_O_6_.^25^ Owing to the high coordination with linkers, Zr‐based MOFs generally possess good moisture and chemical stabilities.^26^, ^27^ Besides, the pore and particle sizes of Zr‐based MOFs can be tuned by varying the linkers and SBUs.^28^ Therefore, they have been widely employed for gas separation,^29^ catalysis,^30^ and even the drug delivering.^31^

MOFs have also been used in the photovoltaic field. At an earlier time, MOF has been introduced to modify the surface of TiO_2_ electrode in dye‐sensitized solar cells.^32^ Thus far, the employment of MOFs in PVSCs is still rare and most of the reported studies are using MOFs as the charge‐transporting layers (CTLs) in PVSCs. For example, in 2014, Vinogradov et al. first introduced MOFs at the perovskite interface to fabricate the device.^33^ More recently, several groups utilized functional MOFs as the CTLs or as the additive in the CTLs to improve PVSCs' performance as a result of facilitated charge extraction or enhanced light absorption.^34^, ^35^ Interestingly, a few recent studies have revealed that, while using MOF as an interlayer, it could provide additional porous scaffold to promote the perovskite crystallization at initial stage and thus enhance the grain sizes of the prepared film grown on them.^36^

**Figure 1:**
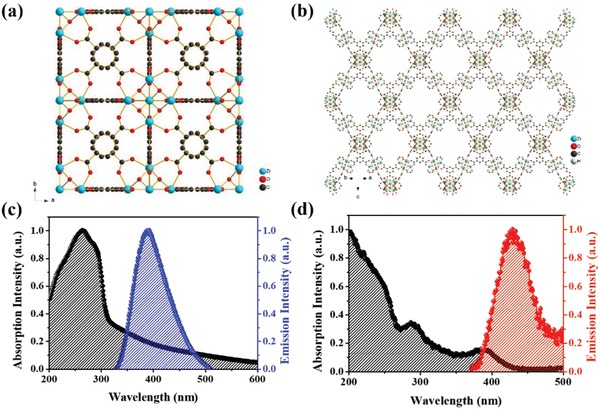
The crystal structures of a) UiO‐66 and b) MOF‐808. The UV–vis absorption and PL spectra of the c) UiO‐66 and d) MOF‐808 films.

In contrast to the employment of MOFs in the CTLs, the perovskite/MOF hybrid heterojunction is rarely investigated and its effectiveness in device fabrication seems not to be fully explored yet although the crystallinity of perovskite film was demonstrated to be enhanced after hybridizing with MOF.^37^ Therefore, we herein systematically investigated the effectiveness of perovskite/MOF heterojunction in the inverted p–i–n PVSCs, which has not been clearly discussed in the literature yet. In this study, two types of Zr‐MOFs, UiO‐66 and MOF‐808, with different physical properties (e.g., pore size, tunnel structure) were employed owing to their respectable moisture and chemical stabilities (**Figure** **1**a,b).^25^ We first explored the effectiveness of these MOFs as the surface modifier for the NiO*_x_* hole‐transporting layer (HTL). They were revealed to enhance the grain size of the perovskite film grown on top and simultaneously facilitate the charge‐extraction efficiency at the perovskite/NiO*_x_* interface. As a result, the best MOF‐modified‐NiO*_x_* PVSC could deliver an enhanced PCE of 17.01% from 15.79% (control device). We next utilized the perovskite/MOF hybrid heterojunction for device fabrication. The hybrid MOFs were found to possibly distribute over the perovskite grain boundaries providing passivation function; meanwhile, their 3D porous architecture accommodates the filling of small perovskite nanocrystals to afford decent charge‐transporting pathways across the MOF scaffolds. More importantly, as benefitting from superior stability of MOFs, the ambient stability of the hybrid films was largely improved. Consequently, the best MOF‐hybrid PVSC could deliver a further enhanced PCE of 18.01% along with much improved ambient stability.

## 2 Results and Discussion

### 2.1 Synthesis and Characterization

In this study, two kinds of Zr‐MOFs, UiO‐66 and MOF‐808, were employed to modify the crystallinity of perovskite film. The structures of the studied MOFs were illustrated in Figure 1a,b. The UiO‐66 with a formula of Zr_6_O_4_(OH)_4_(BDC)_6_ consists of a SBU of Zr_6_O_4_(OH)_4_(—CO_2_)_12_ and an organic linker of terephthalic acid (H_2_BDC), while the MOF‐808 with a formula of Zr_6_O_4_(OH)_4_(BTC)_2_(HCOO)_6_ comprises a SBU of Zr_6_O_4_(OH)_4_(—CO_2_)_6_ and an organic linker of trimesic acid (H_3_BTC).^25^ Both of them were synthesized according to the procedures reported in the literature with slight modifications.^25^, ^38^ The acetic acid was introduced into the precursor solution of UiO‐66 as a modulator for controlling the growth rate of UiO‐66, while formic acid was introduced into precursor solution of MOF‐808 as a modulator. According to previous report, the modulators could compete with linkers to coordinate with SBUs, which would slow down the crystallization speed of MOF seeds.^39^

The X‐ray diffraction (XRD) characteristics of the synthesized UiO‐66 and MOF‐808 powders were presented in Figure S1a,d (Supporting Information), respectively, wherein all the main peaks are in well congruence with the standard values. Their pore size distribution can be derived from their corresponding nitrogen adsorption–desorption isotherms, as shown in Figure S1b,e (Supporting Information). The two pore sizes (≈0.8 nm and ≈1.3 nm) were obtained for the synthesized UiO‐66 powders; one of which is the window size while the other is the cage size. Whereas, only one pore size of 1.9 nm was observed for the synthesized MOF‐808 powders owing to the different linkers compared to UiO‐66. The specific surface area calculated with Brunauer–Emmett–Teller (BET) theory of UiO‐66 and MOF‐808 was 800 and 854 m^2^ g^−1^, respectively. Notably, the pore sizes for both MOFs are large enough to accommodate the penetration of perovskite precursor, which might facilitate the miscibility/compatibility between perovskite and themselves.^37^

The UV–vis absorption spectra of the UiO‐66 and MOF‐808 films were displayed in Figure 1c,d, wherein both MOFs showed intense absorption in the ultraviolet (UV) region (200–400 nm). Their corresponding photoluminescence (PL) spectra were also showed in Figure 1c,d, in which the maximum PL emission for UiO‐66 and MOF‐808 was at ≈392 and ≈429 nm, respectively. It has been widely discussed that the organic linkers absorbed energy from light and the absorption wavelength of BDC would have redshift as more moieties on the benzene ring.^40^ As described previously, UiO‐66 had a BDC as the organic linker while the organic linker of MOF‐808 contains an additional carboxylic group, resulting in a more red‐shifted absorption band edge as illustrated in Figure 1d. The photoexcited electrons on the organic linkers were then injected into SBUs and separated from holes via a ligand to cluster charge transfer process.^41^, ^42^ The separated electron and charge pair would decay in the microsecond time, generating a fluorescence after the electrons flew back to the organic linkers.^43^ Alvaro et al.^44^ further demonstrated the phenomena of electron transfer between the organic linkers and SBUs by observing the fluorescence from the excited BDCs which would be quenched by addition of zinc ions in the solution.

It should be noted that the optical properties of both UiO‐66 and MOF‐88 are quite suitable for applications in PVSCs since they might provide a down‐conversion of high‐energy photons for the perovskite film.^45^, ^46^, ^47^, ^48^ As seen, their intense absorption in the UV region can help filter the UV radiation, which has been cited to be harmful for perovskite materials. Meanwhile, as inferred from the PL spectra of MOF, if it possesses a decent PL intensity and quantum yield, Förster energy transfer between itself and perovskite could be existed, which is beneficial for improving the resultant photocurrent of derived devices. Therefore, we considered that MOFs possess promising potential to couple with the perovskite materials and thus conducted a systematic investigation of perovskite/Zr‐MOF heterojunctions in PVSCs including the bilayer architecture and the hybrid form.

### 2.2 Using MOF as the Surface Modifier of NiO*_x_* HTL in p–i–n PVSC

To test the effectiveness of perovskite/Zr‐MOF heterojunction, we first inserted the MOFs at NiO*_x_*/perovskite interface, for which the MOFs served as a surface modifier of the NiO*_x_* HTL to modulate the crystallization of the perovskite film grown on top. It has been demonstrated in the literature that this additional MOF scaffold could promote the perovskite nucleation during the film evolution; meanwhile, the polar groups of the MOF structure might temporarily coordinate with Pb^2+^ to modulate the crystallization rate to result in enlarged grain size of the prepared film.^34^, ^35^, ^36^, ^49^ To explore this, the Fourier‐transform infrared spectroscopy of the hybrid perovskite/Zr‐MOF films was measured.^50^, ^51^ To better probe the interaction between MOFs and perovskite, the blended amount of MOFs was increased herein compared to the real case of the hybrid film for device fabrications. As presented in Figure S2 (Supporting Information), the characteristic peak of C—O bonding belonging to the pristine MOF appeared in the MOF/perovskite hybrid film accompanied with slight shift. Besides, the N—H bonding belonging to perovskite was also slightly shifted after blending with MOFs. The observed shift of these polar groups clearly indicates certain interactions between them, which could modulate associated crystal growth of perovskite during film evolution.

Presented in Figure S3 (Supporting Information) are the XRD patterns of the perovskite films grown on the neat NiO*_x_* film and MOF‐modified NiO*_x_* films. As seen, the characteristic peaks of the film grown on the MOF‐modified NiO*_x_* film possessed an increased intensity compared to those of the film grown on the pristine NiO*_x_* film. The full width at half maximum (FWHM) values of the perovskite film grown on the UiO‐66‐ and MOF‐808‐modified NiO*_x_* film are 0.301° and 0.281°, respectively, which are smaller than the value (0.311°) of the film grown on the NiO*_x_* film. This result clearly reveals the improved crystallinity of perovskite film promoted by the MOF modification. The surface morphology of the perovskite film deposited on the MOF‐modified NiO*_x_* film was next investigated using field‐emission gun scanning electron microscope (FEG‐SEM). As displayed in Figure S4 (Supporting Information), the grain size of the perovskite films grown on the MOF‐modified NiO*_x_* films becomes larger compared with the film deposited on the pristine NiO*_x_* film, raising from ≈480 to 720/640 nm for the UiO‐66/MOF‐808‐modified samples, respectively, being consistent with the results reported in the literature.^34^, ^35^, ^36^, ^49^ This result clearly manifests the positive impact of the additional MOF scaffold on improving the crystallinity and grain size of the deposited perovskite film. That said, the defect density and the grain boundaries inside the film can be reduced to promote the charge transfer at associated interface.

**Figure 2:**
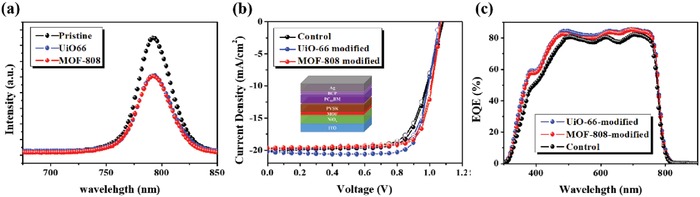
a) The PL spectra of the pristine perovskite film and bilayer MOF/perovskite films. b) The *J*–*V* curves of the MOF‐modified devices measured under 1 sun and c) their corresponding EQE spectra.

To probe this, the PL spectra of these samples were measured as shown in **Figure** **2**a. A certain degree of PL quenching was observed for the perovskite films grown on the studied MOF layers. Such PL quenching can be roughly interpreted as a consequence of the facilitated charge transfer at the perovskite/MOF interface. This enhancement could be attributed to the 3D porous scaffold of MOF, which allows the filling of perovskite precursors and the formation of perovskite nanocrystals to improve the interfacial compatibility and the charge extraction efficiency.^33^, ^34^, ^35^

With these encouraging results, we next fabricated inverted p–i–n PVSCs with a configuration of indium tin oxide (ITO)/NiO*_x_*/MOF/CH_3_NH_3_PbI_3_/PC_61_BM/BCP/Ag. Their current density–voltage (*J*–*V*) characteristics measured under AM 1.5 G solar irradiance (100 mW cm^−2^) were presented in Figure 2b and the relevant photovoltaic parameters including open‐circuit voltage (*V*_oc_), short‐circuit current (*J*_sc_), and fill factor (FF) were summarized in **Table** **1**. All the fabricated devices were revealed to possess negligible hysteresis. Further, it can be seen that the slope of the curve nearby the *V*_oc_ for the MOF‐modified PVSCs is higher than that of the control device, suggesting the reduced series resistance in the former devices. This confirms that the MOF interlayer improves the electrical contact at the perovskite/NiO*_x_* interface, which could be resulted from the improved crystallinity of perovskite films and the improved interfacial compatibility as discussed earlier.

**Table 1:** The photovoltaic parameters of the MOF‐modified PVSCs

	*V*_oc_ [V]	*J*_sc_ [mA cm^−2^]	FF [%]	PCE [%]
PVSCa)	1.060	19.23	77.5	15.79
MOF‐808 modifiedb)	1.068	19.64	78.9	16.55
UiO‐66 modifiedb)	1.067	20.25	78.5	17.01
MOF‐808 hybridc)	1.062	21.01	79.8	17.81
UiO‐66 hybridc)	1.072	21.85	76.9	18.01

^a)^ Control device

^b)^ MOFs as the surface modifier of NiO*_x_*

^c)^ MOF/perovskite hybrid.

This improvement leads to the enhanced PCEs of both MOF‐modified PVSCs. The MOF‐808/UiO‐66‐modified PVSC delivered a PCE of 16.55% and 17.01%, respectively, surpassing the performance (15.79%) of the control device. The enhanced PCEs were mainly contributed from the increases in *J*_sc_ and FF. The increased FF was attributed to the improved interfacial electrical contact as mentioned, while the improved *J*_sc_ was ascribed to the improved light‐harvesting capability of the perovskite film owing to the promoted crystallization (Figure S5b, Supporting Information). On the other hand, the possible energy transfer from UiO‐66 to perovskite (Figure 1c; Figure S5a, Supporting Information) might further contribute to the increased photocurrent, as reflected in its highest photocurrent (20.25 mA cm^−2^) among the fabricated devices.

To analyze the photoresponse of the fabricated devices, the external quantum efficiency (EQE) spectra of the fabricated devices were recorded using a AM 1.5G reference spectrum as presented in Figure 2c. All the *J*_sc_s integrated from the spectra well matched the values obtained in the *J*–*V* measurement, confirming the accuracy of device measurement. As seen, both MOF‐modified PVSCs possessed enhanced photoresponse across 300–780 nm compared to the control device. As being consistent to the enhanced absorption (Figure S5b, Supporting Information), the photoresponse from 300–500 nm region was greatly improved. We further measured the internal quantum efficiency (IQE) to clarify the enhanced EQE in this region (300–500 nm). As shown in Figure S6a (Supporting Information), the IQE for both MOF‐modified devices was increased in the region of 300–500 nm compared to the control device, suggest the nontrivial role of MOF in contributing to this. Therefore, besides the improved absorption of perovskite layer or the interference effects, the energy transfer between MOF and perovskite might also be considered (Figure 1c,d), particularly for the case of UiO‐66 that showed intense UV absorption and PL emission (Figure S5a, Supporting Information). It definitely warrants more in‐depth investigation in the future but this result reveals that using MOF as the interlayer at the light incoming side might possess potential advantage of converting the UV radiation to simultaneously increase the photocurrent and UV stability of the derived devices.^45^, ^46^, ^47^, ^48^

To further verify the defect passivation, we investigated the trap density of the studied perovskite films by fabricating hole‐dominated devices with a device configuration of ITO/NiO*_x_*/MOFs/perovskite/MoO_3_/Ag.^52^, ^53^, ^54^ The *J*–*V* measurements of these devices were measured under a dark condition, as presented in Figure S7a (Supporting Information). In principle, three main regions in the corresponding *J*–*V* curves were generally identified according to slope's change: ohmic contact, trap‐filled limit (TFL) current, and space‐charge‐limited current. Based on the TFL current, the trap density could be estimated using the equation of *V*_TFL_ = *en*_t_ *L* ^2^/2*εε*_0_, where *V*_TFL_ is the trap‐filled limit voltage, *e* is the elementary charge, *n*_t_ is trap density, *L* is the thickness (600 nm), and ε and ε_0_ are the vacuum permittivity and the dielectric constants of MAPbI_3_. The estimated trap density is 2.26 × 10^15^ cm^−3^ for the UiO‐66‐modified device, 3.25 × 10^15^ cm^−3^ for the MOF‐808‐modified device, and 5.51 × 10^15^ cm^−3^ for the control device, respectively. The reduced trap density observed in the MOF‐modified device manifested the possible trap passivation enabled by the MOF interlayer.

### 2.3 Fabricating p–i–n PVSC Using Perovskite/MOF Hybrid Heterojunction

**Figure 3:**
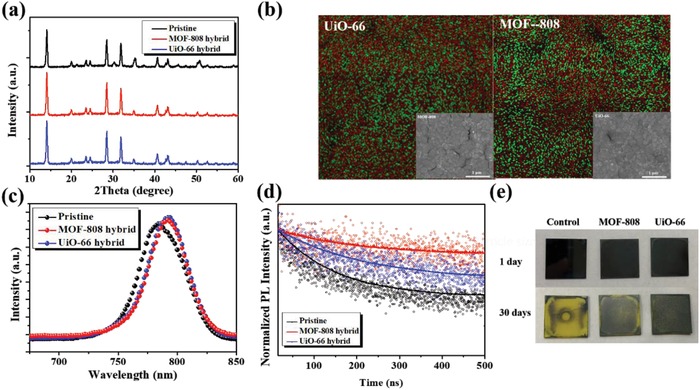
a) The XRD patterns, b) the SEM‐EDS images (red is I and green is Zr), c) the PL spectra, and d) time‐resolved PL spectra of the studied MOF‐hybrid films. e) The real‐time images of the hybrid film stored in ambient condition (25 °C and RH: 60 ± 5%).

Based on this encouraging result, we next attempt to utilize perovskite/MOF hybrid heterojunction to fabricate PVSCs. Prior to device fabrication, we first explore the properties of the hybrid films and the detailed preparation of the films was described in the Experiment Section. **Figure** **3**a presents the XRD patterns of the pristine perovskite film and the studied hybrid films. As seen, the characteristics belong to the perovskite phase can be clearly traced, in which the crystalline peaks of MOFs were overshadowed due to its limited amount in the hybrid films.

The surface morphology of the hybrid films was next examined by SEM as shown in the insets of Figure 3b, wherein a compact, smooth film with densely packed morphology was observed for both hybrid films. The absence of severe phase separation suggests the reasonable miscibility between them. We further checked the Zr/I element distribution using scanning electron microscopy‐energy dispersive spectroscopy (SEM‐EDS) to better probe the distribution of MOF in the hybrid film. As shown in Figure 3b, unlike the homogeneous distribution of I atoms, the Zr atoms show a slight inhomogeneous distribution in the film. Such discrepancy in the spatial distribution plausibly indicates the possibility of MOF to distribute over the perovskite grain boundaries. Since the electron lone pairs from oxygen inside the MOF structure could coordinate with the Pb^2+^ ions, function of defect passivation could be envisioned.

To confirm this effect, the PL spectra of these studied films were measured. As shown in Figure 3c, the PL intensity of the hybrid films was higher than the pristine perovskite film. Such increase implies a certain degree of defect passivation induced by the hybrid MOFs, different to the previous case of bilayer heterojunction. We notice that the PL emission peak of the hybrid films was slightly red‐shifted compared to the pristine film (Figure S8, Supporting Information). The clear reason is not readily understood at this stage and warrants further investigations. However, we believe it is related to the interactions between MOFs and perovskite. It can be observed that the hybrid films (insets in Figure 3b) possessed smaller grain size (≈240–300 nm) than the pristine film (480 nm), manifesting the critical role of the interaction between them. Different to the previous case using MOF as an interlayer, the direct hybridization with MOF seems to slightly restrain the grain growth of perovskite; however, the overall influence is not that profound. Time‐resolved photoluminescence measurement was further conducted to probe the charge dynamics of these studied films. Their corresponding PL decay kinetics were illustrated in Figure 3d and the fitted lifetimes for the pristine film, MOF‐808 hybrid film, and UiO‐66 hybrid film are 133.46, 194.50, and 202.55 ns, respectively. Both the hybrid films exhibited a slower PL decay compared to the pristine film, suggesting the retarded charge recombination inside the film. These results confirm the defect annihilation ability of the hybrid MOFs. Furthermore, such effect could be also verified by the lower trap density in its corresponding hole‐dominated device. As calculated from the results shown in Figure S7b (Supporting Information), the trap density for the UiO‐66‐ and MOF‐808‐hybrid devices is 4.92 × 10^15^ and 2.66 × 10^15^ cm^−3^, which is lower than the value (5.51 × 10^15^ cm^−3^) of the control device. This result clearly reveals the defect passivation function of the hybrid MOF.

Another benefit of the passivation function imposed by the hybrid MOFs is to prohibit the moisture diffusions through grain boundaries into the bulk film; meanwhile, the incorporated MOF themselves possess good moisture stability. Therefore, we next traced the ambient stability of the hybrid films along with the pristine perovskite film. The stability test was conducted in ambient air at room temperature with a relative humidity (RH) of 60 ± 5% and all the studied perovskite films were coated on glass substrates without any surface protection. As presented in Figure 3e, both hybrid films showed a much retarded degradation compared to the pristine film. The color of the pristine film almost faded after 30‐day storage, whereas the hybrid films still possessed original dark brown color after the same time storage. This discrepancy clearly manifests the advantages of hybridizing MOF into the perovskite films. The MOF distributing over the grain boundaries might wrap the grain to provide plausible grain‐locking effect to reinforce the film robustness against the moisture invasion.

**Figure 4:**
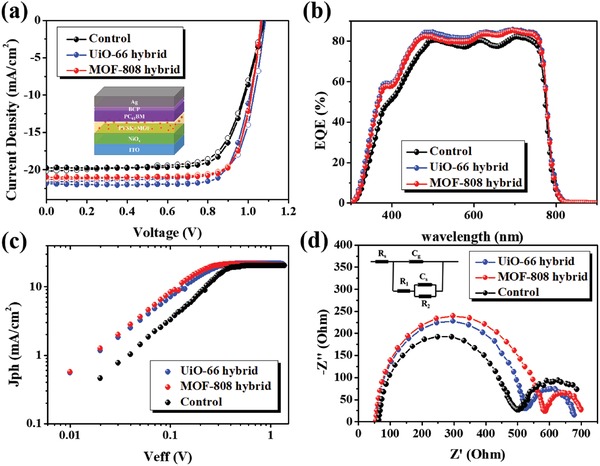
a) The *J*–*V* curves, b) the EQE spectra, c) *J*_ph_–*V*_eff_ characteristics, and d) the EIS analysis of the studied MOF‐hybrid PVSCs.

We finally fabricated the inverted PVSCs using these perovskite/MOF hybrid films. Their corresponding *J*–*V* curves measured under AM 1.5 G solar irradiance (100 mW cm^−2^) were presented in **Figure** **4**a and the resultant performance was summarized in Table 1. As seen, both MOF‐hybrid PVSCs delivered comparable *V*_oc_ and FF to the value of control device. This result validates that the hybridization of MOF does not significantly affect the electronic property of the perovskite film. It can be envisioned that their porous architecture accommodates the filling of small perovskite nanocrystals to afford decent charge‐transporting pathways across the MOF scaffolds. Nevertheless, after hybridization with MOF, the resultant *J*_sc_ was increased. The *J*_sc_s for the control, MOF‐808 hybrid, UiO‐66 hybrid devices were 19.23, 21.01, and 21.85 mA cm^−2^, respectively. The increased *J*_sc_s thus enable the MOF‐808 hybrid and UiO‐66 hybrid devices to possess a promising PCE of 17.81% and 18.01%, respectively, representing a 13–14% enhancement in performance compared to the control device.

To confirm the increased photocurrents, the EQE spectra of these fabricated devices were taken as displayed in Figure 4b. Again, all the *J*_sc_s integrated from the spectra well matched the values obtained in the *J–V* measurement, confirming the accuracy of device measurement. Similar to the previous case of using studied MOFs as the interlayers, the photoresponse across 300–500 nm was clearly increased for both MOF‐hybrid devices (Figure S6b, Supporting Information). Again, it could be resulted from the improved absorption of perovskite film or the possible energy transfer between MOF and perovskite as discussed earlier. Similarly, the UiO‐66 hybrid device delivered the highest photocurrent (21.85 mA cm^−2^) among the fabricated devices owing to its more intense absorption in the UV region (Figure S3a, Supporting Information) and possible energy transfer to the perovskite (Figure 1c).

The photoexcitation behavior of the fabricated devices was further analyzed by plotting the photocurrent density (*J*_ph_)–effective voltage (*V*_eff_) as portrayed in Figure 4c. The *J*_ph_ is defined as *J*_ph_ = *J*_L_ −*J*_D_, wherein *J*_L_ is the photocurrent measured under 1 sun AM 1.5G spectrum and *J*_D_ is the dark current measured under a totally dark condition. The *V*_eff_ is defined as *V*_eff_ = *V*_0_ − *V*_bias_, for which *V*_0_ is the voltage when *J*_ph_ = 0 and *V*_bias_ is the applied bias. In principle, *J*_sat_ can be defined as the current where all the generated photoexcitons were dissociated into free carriers. The estimated *J*_sat_ for the control, MOF‐808 hybrid, and UiO‐66 hybrid devices are 20.37, 21.04, and 21.23 mA cm^−2^, respectively. The maximum photoexciton generation rate (*G*_max_) thus can be calculated by the equation of *J*_sat_ = *eG*_max_ *L*, where *e* represents the elementary charge and *L* is the thickness of the active layer (600 nm herein). The calculated *G*_max_ for the control, MOF‐808 hybrid, and UiO‐66 hybrid devices are 2.12 × 10^27^, 2.19 × 10^27^, and 2.21 × 10^27^ s^−1^ m^−3^, respectively. The higher *G*_max_ represents the better utilization of the generated photoexcitons, affirming the increased *J*_sc_ observed in the MOF‐hybrid devices. Meanwhile, the charge collection efficiency can be estimated by charge collection probability (*P*), which is calculated by the equation of *P* = *J*_ph_/*J*_sat_. For instance, when setting *V*_eff_ = 0.3 V, the calculated *P* for the control, MOF‐808 hybrid, and UiO‐66 hybrid devices is 73.4%, 98.3%, and 92.6%, respectively. This result unveils the improved charge collection efficiency of the MOF‐hybrid devices, which might be beneficial from the defect passivation effects introduced by them as previously discussed.^34^, ^35^

To understand the electrical feature of the fabricated PVSCs, the electrochemical impedance spectroscopy (EIS) measurements were conducted in ambient environment at 0.8 V under 1 sun illumination. Shown in Figure 4d are their corresponding Nyquist plots, wherein two semi‐circles were showed. The figure in inset is the matryoshka equivalent circuit used for data fitting. The series resistance (*R*_s_) accounts for the ohmic contribution of external contact and was not coupled with any capacitance. *R*_1_ and *C*_g_ represent the high‐frequency response, where *C*_g_ is the geometric capacitance related to the dielectric property of the perovskite layer and *R*_1_ relates to the transport resistance of electrons within the perovskite layer. *R*_2_ and *C*_s_ are observed at the low‐frequency arc, where *C*_s_ represents the ionic accumulation capacitance in dark and the charge accumulation capacitance in light while *R*_2_ coupled with *R*_1_ stands for the resistance of surface recombination (*R*_rec_).^55^, ^56^, ^57^

We herein focus on the analyses of the resistance of surface recombination (*R*_rec_) and the dielectric properties of bulk perovskite layer. **Table** **2** and Table S1 (Supporting Information) summarized the fitting results, wherein *R*_rec_ = *R*_1_ + *R*_2_. The *R*_rec_ for both MOF‐hybrid devices is much higher than the value of control device, indicating that the hybridization with MOFs could relieve the charge recombination in device to result in better photovoltaic performance. Further, in Table S1 (Supporting Information), both the MOF‐hybrid devices were shown to possess a lower *C*_g_ than the value of control device. While *C*_g_ is mainly associated with the geometry of perovskite layer, the orientation of CH_3_NH_3_^+^ ions or PbI_2_, and the cooperative ionic off‐center,^55^ the smaller values indicate that the hybridization with MOFs could surely enhance the crystallization of perovskite layer.

**Table 2:** The analyzed resistance of the studied MOF‐hybrid PVSCs

	*R*_s_ [Ohm]	*R*_1_ [Ohm]	*R*_2_ [Ohm]
None	36.41	385.4	190.1
MOF‐808a)	45.02	538.4	120.3
UiO‐66a)	60.01	463.8	148.2

^a)^ MOF/perovskite hybrid device.

**Figure 5:**
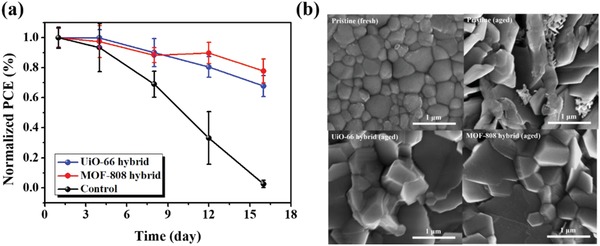
a) The PCE of the fabricated devices as a function of storage time in ambient air (25 °C and RH: 60 ± 5%) and b) the surface SEM images of the studied films after 30‐day aging time.

Provided the improved ambient stability of the MOF‐hybrid films, we finally examine the ambient stability of the fabricated devices. Present in **Figure** **5**a are their PCE as a function of the storage time in ambient air at room temperature with a RH of 60 ± 5% without any encapsulation. As seen, ≈70% and ≈80% of initial performances were respectively retained for the UiO‐66 hybrid and MOF‐808 hybrid devices after two‐week aging time, whereas the PCE of the control PVSC was dramatically dropped to merely 3%. This result clearly reflects the effectiveness of using perovskite/MOF hybrid heterojunction in enhancing the overall ambient stability. To further trace the underlying mechanism, the surface morphology of the aged films was examined by SEM. As shown in Figure 5b, the perovskite grains in the MOF‐hybrid films were better preserved compared to the pristine film. For both hybrid films, the compact grain texture was still maintained after 30‐day aging time, which could still provide reasonable charge transport to result in reasonable performance. By contrast, discrete texture was formed in the pristine perovskite film after same aging time, which thus results in the poor performance as observed. This result unveils the plausible grain‐locking effect of the hybrid MOFs to reinforce the film robustness against the moisture invasion.

## 3 Conclusion

In summary, we herein described the effectiveness of perovskite/Zr‐MOF heterojunction, including bilayer architecture and the hybrid form, in fabricating high‐performance inverted p–i–n PVSCs. Two types of Zr‐MOFs, UiO‐66 and MOF‐808, were first employed as the surface modifier for the NiO*_x_* HTL in the device and were shown to enhance the crystallization of the perovskite film grown on top and simultaneously facilitate the charge‐extraction efficiency at the corresponding interface. Consequently, the UiO‐66/MOF‐808‐modified PVSCs could yield enhanced PCEs of 17.01% and 16.55%, outperforming the control device (15.79%). Moreover, we further exploited the perovskite/Zr‐MOF hybrid heterojunction to fabricate the devices. The hybrid MOFs were found to possibly distribute over the perovskite grain boundary, providing the grain‐locking effect. It not only passivates the defects but also reinforces the film's robustness against the moisture invasion. The PCEs of the UiO‐66/MOF‐808‐hybrid PVSCs could be further enhanced to 18.01% and 17.81%, respectively. More intriguingly, over 70% of initial PCE can be retained after being stored in ambient air (25 °C and RH of 60 ± 5%) for over 2 weeks in contrast to the quick degradation observed for the control device. This study unravels the effectiveness of using perovskite/MOF heterojunction to fabricate efficient and stable solar cells, providing a new strategy for the future design of PVSCs.

## 4 Experimental Section

*Materials*: The studied MOFs, UiO‐66 and MOF‐808, were prepared according to the procedures reported in the literature with slight modifications.^25^, ^38^ For synthesis of UiO‐66, zirconium tetrachloride (40 µmol) was dissolved in 5 mL of dimethylformamide (DMF) and BDC (40 µmol) was dissolved in 5 mL of DMF containing acetic acid (4.8 m). The zirconium tetrachloride‐containing DMF was poured into organic linker‐containing DMF solution in a Teflon container. The Teflon container was sealed in a Parr reactor and reacted at 120 °C for 8 h. The white UiO‐66 sample was spun down by centrifugation at 10 000 r.p.m for 10 min and washed with DMF for two times and methanol for three times. The as‐synthesized UiO‐66 was dried at 120 °C overnight to remove rest DMF before usage. For synthesis of MOF‐808, zirconyl chloride octahydrate (0.5 mmol) and BTC (0.5 mmol) were dissolved in a mixture containing 20 mL of DMF and 20 mL of formic acid. The solution was sealed in a glass vail at 100 °C for 7 days. The white MOF‐808 particles were collected by centrifugation and washed with DMF for one time. After that, the collected particles were suspended in solution to remove free linkers for six days. The solution was fresh DMF in first three days and replaced with anhydrous acetone in last three days. The as‐synthesized MOF‐808 was further dried at 150 °C for one day before usage.

The precursor solution of the NiO*_x_* HTL was prepared by dissolving nickel(II) 2,4‐pentanedionate powders in ethanol with a concentration of 25.7 mg mL^−1^ and addition of HCl (10 µL mL^−1^). The precursor solution of regular MAPbI_3_ perovskite (denoted as precursor A) was prepared by dissolving PbI_2_ and MAI in a mixed solvent of dimethyl sulfoxide (DMSO) and DMF (1:4 v/v) at a molar ratio of 1:1 (with a targeted concentration for 1.2 m MAPbI_3_) and stirred at 60 °C overnight. The difference between the precursor A and the precursor solutions (denoted as precursor B) for the MAPbI_3_/MOF hybrids mainly lies in the solvent content, for which half content of DMF in precursor A was replaced by the MOF suspension solutions (1 mg mL^−1^ in DMF for both UiO‐66 and MOF‐808) to prepare the precursor B.

*Device Fabrication*: The studied PVSCs were fabricated in an inverted p–i–n configuration. The patterned ITO‐coated glasses were cleaned by detergent, DI‐water, acetone, and isopropanol for 15 min sequentially. After drying by nitrogen gas and the further treatment with plasma for 20 min, the NiO*_x_* HTL was spin‐coated onto ITO at 5000 rpm for 10s, followed by annealing at 325 °C in air for 45 min. To avoid moisture and oxygen, the following fabrication procedures were performed in a N_2_‐filled glove‐box. The perovskite active layer was prepared by a three‐step spin‐coating process: 2000 rpm for 10 s, 3000 rpm for 7 s, and 4500 rpm for 20 s sequentially. 450 µL toluene was continuously dripped onto the spin‐coated substrate at the end of second step during the spin‐coating process. For the PVSC using MOF as the interlayer, a MOF (0.5 mg mL^−1^ in DMF) layer was first coated onto the NiO*_x_* HTL at 4000 rpm for 30 s prior to the deposition of perovskite active layer, followed by thermal annealing at 100 °C for 7 min. For the perovskite/MOF device, the perovskite active layer was fabricated using precursor B and followed the same spin‐coating conditions. Afther the deposition of active layers, PC_61_BM (20 mg mL^−1^ in chlorobenzene) and BCP (0.5 mg mL^−1^ in isopropyl alcohol) were sequentially deposited onto the perovskite films with a spin‐coating condition of 2000 rpms for 30 s and 6000 rpm 7 s, respectively. Finally, a 100 nm thick Ag electrode was thermally evaporated under high vacuum (< 4.0 × 10^−6^ Torr) throught a shadow mask. For all the devices, the active area was definied to be 0.1 cm^2^.

*Characterization*: The *J*–*V* curve was recorded with a computer‐controlled Keithley 2400 source under AM1.5G (100 mW cm^−2^) illumination by a Newport LCS‐100 simulator. The EQE was measured with QE‐R, Enlitech Co., Ltd, using AM1.5G reference spectrum and corrected by a single crystal Si photovoltaic cell. The FEG‐SEM and EDS images were taken by NOVA NANO SEM 450. The EIS resistance analysis was measured with Solartron Analytical in ambient environment at 0.8 V under 1 sun illumination, and fitted by using EC lab software. The PL lifetime was recorded with Edinburgh life spec‐1700, and measured by time‐correlated single photo counting. Besides, it was fitted using the equation of *R*(*t*) = *B*_1_exp(−*t*/τ_1_) + *B*_2_exp(−*t*/τ_2_), where *B*_1_ and *B*_2_ are the constants varied with each conditions.

## Conflict of Interest

The authors declare no conflict of interest.

## Supporting information

Supplementary
