# Supplementary material for: Enhancing Efficiency and Stability of Photovoltaic Cells by Using Perovskite/Zr‐MOF Heterojunction Including Bilayer and Hybrid Structures
Source: Adv Sci (Weinh). 2019 Jan 1;6(5):1801715. doi: 10.1002/advs.201801715 (PMC6402453; doi:10.1002/advs.201801715)
Supplement: Supplementary file 1 — Supplementary [file ADVS-6-1801715-s001.pdf]

Copyright WILEY-VCH Verlag GmbH & Co. KGaA, 69469 Weinheim, Germany, 2018.

ADVANCED SCIENCE Open Access

Supporting Information

for *Adv. Sci.,* DOI: 10.1002/advs.201801715

Enhancing Efficiency and Stability of Photovoltaic Cells by Using Perovskite/Zr-MOF Heterojunction Including Bilayer and Hybrid Structures

Chia-Chen Lee, Chih-I Chen, Yu-Te Liao, Kevin C.-W. Wu, and Chu-Chen Chueh**

Copyright WILEY-VCH Verlag GmbH & Co. KGaA, 69469 Weinheim, Germany, 2018.

Supporting Information

Enhancing Efficiency and Stability of Photovoltaic Cells by Using Perovskite/Zr-MOF Heterojunction Including Bilayer and Hybrid Structures

Chia-Chen Lee,[a] *Chih-I Chen,*[a,b] *Yu-Te Liao,*[a] *Kevin C.-W. Wu,*[a,c,d*] *and Chu-Chen Chueh*[a,b,*]

[a] C.-C. Lee, C.-I. Chen, Dr. Y.-T. Liao, Prof. C.-W. Wu, and Prof. C.-C. Chueh
Department of Chemical Engineering, National Taiwan University, Taipei 10617, Taiwan

[b] C.-I. Chen, Prof. C.-C. Chueh
Advanced Research Center for Green Materials Science and Technology, National Taiwan University, Taipei 10617, Taiwan

[c] Prof. Kevin C.-W. Wu
Center of Atomic Initiative for New Materials (AI-MAT), National Taiwan University, Taipei 10617, Taiwan

[d] Prof. Kevin C.-W. Wu
International Graduate Program of Molecular Science and Technology (NTU-MST), National Taiwan University, Taipei 10617, Taiwan

[*]Corresponding author. E-mail: kevinwu@ntu.edu.tw; cchueh@ntu.edu.tw

Keywords: Perovskite; solar cells; metal organic framework; heterojunction; stability

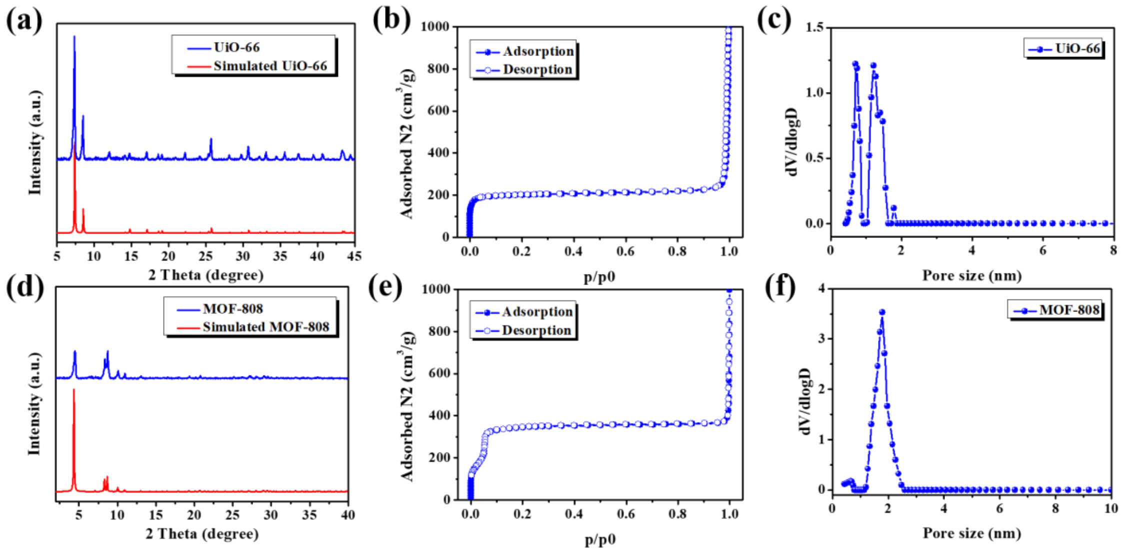

Figure S1. (a, d) The XRD pattern, (b, e) the nitrogen adsorption-desorption isotherms, and (c, f) the pore size analysis of the synthesized UiO-66 and MOF-808 powders.

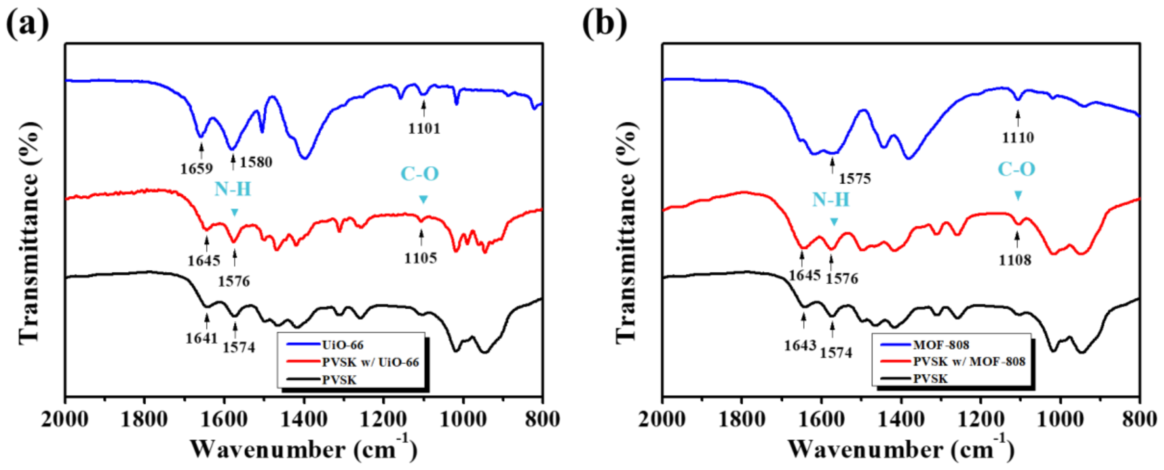

Figure S2. The Fourier-transform infrared spectroscopy (FTIR) of (a) UiO-66/perovskite and (b) MOF-808/perovskite films.

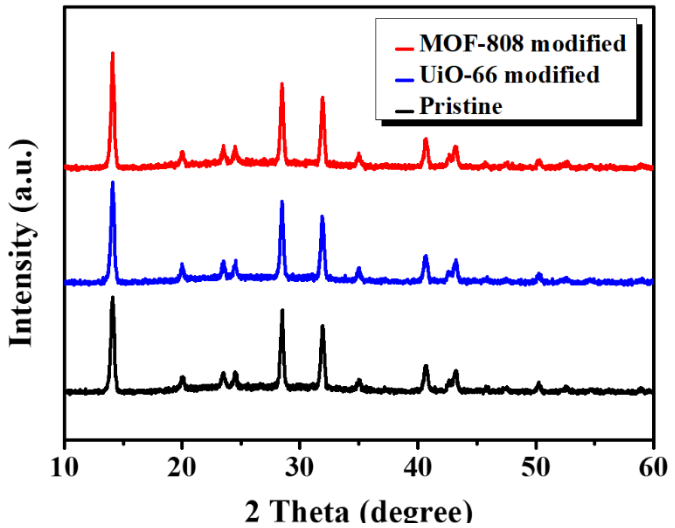

Figure S3. The XRD patterns of the perovskite films grown on neat NiO_x film and MOF-modified NiO_x films.

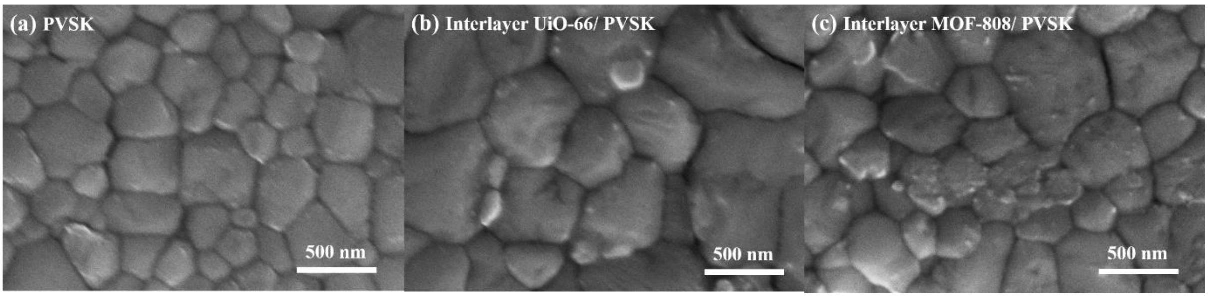

Figure S4. The surface SEM images of the perovskite film grown on the (a) pristine NiO_x film and (b) UiO-66 and (c) MOF-808 modified NiO_x films.

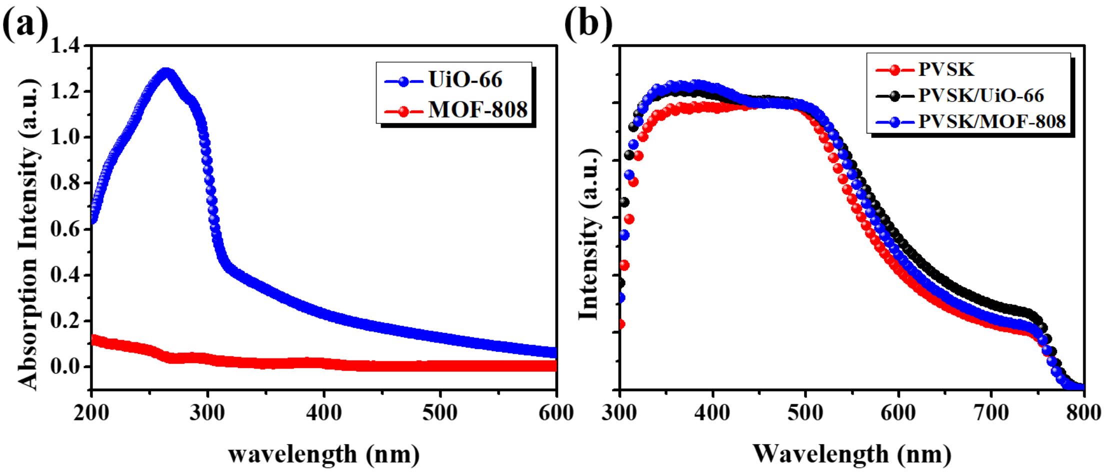

Figure S5. The UV-vis absorption spectra of (a) the MOF films with same thickness without normalization and (b) the pristine perovskite film and the MOF-perovskite bilayer films without normalization, wherein the thickness of the perovskite film is fixed at 600 nm.

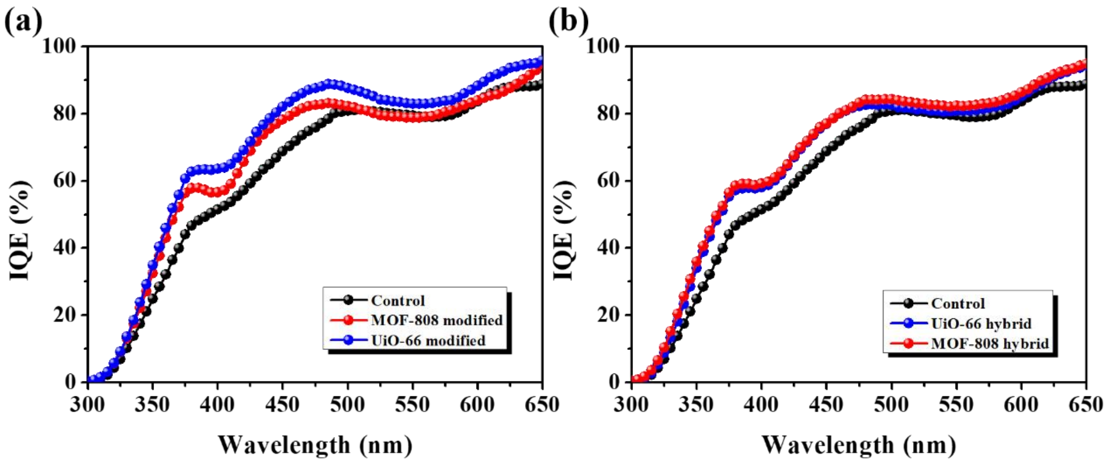

Figure S6. The internal quantum efficiency (IQE) of the MOF-modified and the MOF-hybrid devices.

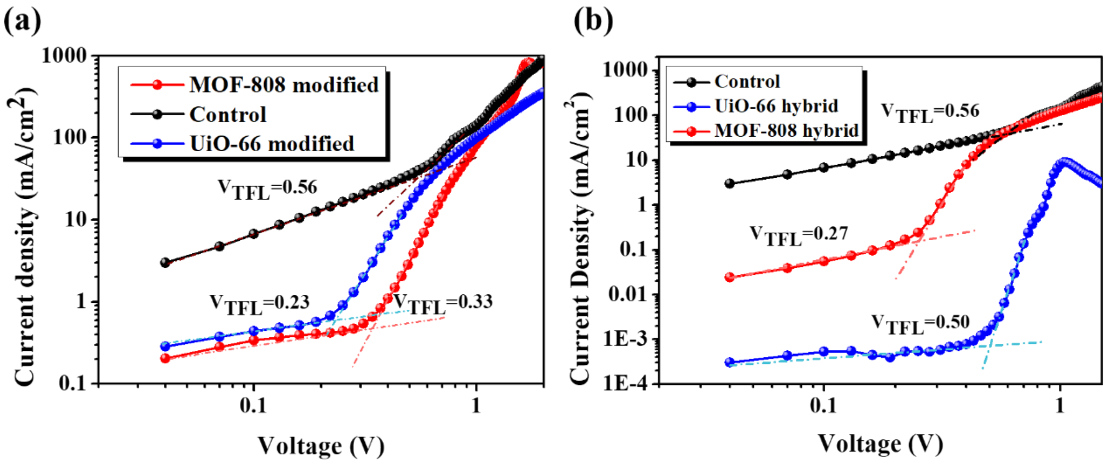

Figure S7. The *J-V* curve of the hole-dominated devices: (a) the case of MOF-modified devices and (b) the case of MOF-hybrid devices.

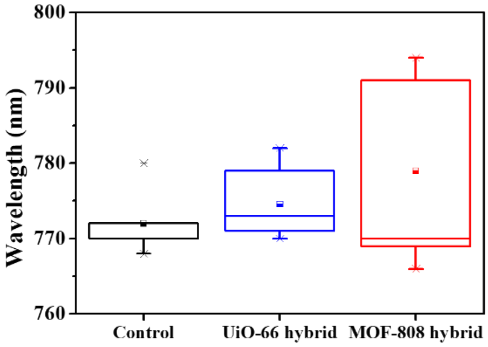

Figure S8. The PL peak distributions of the pristine perovskite, UiO-66/perovskite, and MOF-808/perovskite films.

Table S1. Capacitance parameters in EIS fitting.

	C_g (F.s/cm^2)	C_s (F.s/cm^2)
**PVSC**	3.27×10^{-8}	1.151×10^{-4}
**MOF-808 hybrid**	2.04×10^{-8}	1.017×10^{-4}
**UiO-66 hybrid**	6.88×10^{-9}	3.542×10^{-5}
